# Biomineralization in Three-Dimensional Scaffolds Based on Bacterial Nanocellulose for Bone Tissue Engineering: Feature Characterization and Stem Cell Differentiation

**DOI:** 10.3390/polym15092012

**Published:** 2023-04-24

**Authors:** Ana Cañas-Gutiérrez, Lenka Toro, Cristina Fornaguera, Salvador Borrós, Marlon Osorio, Cristina Castro-Herazo, David Arboleda-Toro

**Affiliations:** 1 Research Group on New Materials (GINUMA), Faculty of Engineering, Universidad Pontificia Bolivariana, Circular 1 No. 70-01, Medellín 050031, Colombia; 2 Biomedical Engineering Research Group (GIBEC), EIA University, Km 2 + 200 on the Way to the José María Córdova Airport, Alto de Las Palmas, Envigado 055428, Colombia; 3 Cancer Research Institute, Biomedical Research Center, University Science Park for Biomedicine, Slovak Academy of Sciences, 84505 Bratislava, Slovakia; 4 Grup d’Enginyeria de Materials (Gemat), Institut Químic de Sarrià (IQS), Universitat Ramon Llull (URL), Via Augusta 390, 08017 Barcelona, Spain; 5 Group of Biosocial Studies of the Body-EBSC-, Faculty of Dentistry, Universidad de Antioquia Calle 64 No. 52-59, Medellín 050010, Colombia

**Keywords:** nanocomposites, bacterial nanocellulose, microporosity, biomineralization and bone regeneration

## Abstract

Bacterial nanocellulose (BNC) has a negative surface charge in physiological environments, which allows the adsorption of calcium ions to initiate the nucleation of different calcium phosphate phases. The aim of this study was to investigate different methods of mineralization in three-dimensional microporous bacterial nanocellulose with the intention of mimicking the composition, structure, and biomechanical properties of natural bone. To generate the 3D microporous biomaterial, porogen particles were incorporated during BNC fermentation with the *Komagataeibacter medellinensis* strain. Calcium phosphates (CPs) were deposited onto the BNC scaffolds in five immersion cycles, alternating between calcium and phosphate salts in their insoluble forms. Scanning electron microscopy (SEM) showed that the scaffolds had different pore sizes (between 70 and 350 µm), and their porous interconnectivity was affected by the biomineralization method and time. The crystals on the BNC surface were shown to be rod-shaped, with a calcium phosphate ratio similar to that of immature bone, increasing from 1.13 to 1.6 with increasing cycle numbers. These crystals also increased in size with an increasing number of cycles, going from 25.12 to 35.9 nm. The main mineral phase observed with X-ray diffraction was octacalcium dihydrogen hexakis phosphate (V) pentahydrate (OCP). In vitro studies showed good cellular adhesion and high cell viability (up to 95%) with all the scaffolds. The osteogenic differentiation of human bone marrow mesenchymal stem cells on the scaffolds was evaluated using bone expression markers, including alkaline phosphatase, osteocalcin, and osteopontin. In conclusion, it is possible to prepare 3D BNC scaffolds with controlled microporosity that allow osteoblast adhesion, proliferation, and differentiation.

## 1. Introduction

Tissue engineering seeks to create microenvironments that activate the self-cells of patients to promote regeneration through the development of new biomaterials. This field uses biomimetics as a strategy to develop scaffolds that mimic the extracellular matrix (ECM) of native tissues, thus improving the biological affinity between cells and the materials that serve as substrates [1,2]. Regarding the ECM of bone tissue, it is important to emphasize that bone is composed of two phases—an organic and an inorganic phase—both fulfilling a specific function in tissue homeostasis. For instance, the mechanical performance of the organic phase composed of type I collagen and other proteins imparts flexibility to the tissue, while the inorganic phase composed of calcium phosphates provides rigidity [3]. In terms of the compositional and biological characteristics of bone, it is important to note that bone is a highly dynamic tissue with a composition that varies considerably during the bone maturation process and constantly during the bone remodeling process [4,5,6].

Therefore, when designing a bone graft, the biomaterial used must activate the mechanisms that underpin the bone repair and regeneration process, including osteoinduction, osteoconduction, and osteogenesis [7,8]. Thus, the new graft must stimulate the osteoinduction process through the differentiation of mesenchymal stem cells (MSCs) into osteoblasts, which are mature bone-forming cells. Additionally, the material should promote osteogenesis through the formation of new bone at the injury site [9,10]. Finally, the bone graft must have a microporous three-dimensional structure that provides an osteoconductive scaffold to guide bone growth, allowing vascular invasion and cellular infiltration within the interconnected pores [11].

Currently, autologous bone grafts, allografts, and xenografts are the most commonly used methods for the regeneration of bone structures [12,13,14]. However, due to the disadvantages of the available bone grafts, including the invasiveness of treatments and immune-rejection responses, the search for new alternatives through the design of biomaterials that can be used as grafts or bone substitutes is still ongoing. Biodegradable natural polymers are very popular in bone tissue engineering [1]; however, although they allow cell proliferation, they have rapid reabsorption rates, which prevent cells from properly forming new tissue [15]. This is a current medical need that bacterial nanocellulose (BNC) can address, as it is a natural polymer that is not biodegradable in vivo [16,17]. BNC possesses the physical, chemical, and biocompatibility properties required to restore normal tissue function and can be used as a replacement for the matrix (scaffold) [18].

One of the most attractive features of BNC is its unique nanofibrillary structure that mimics the structure of the native extracellular matrix, allowing rapid tissue healing and regeneration [19]. In addition, BNC has high reproducibility and does not contain any components of animal origin that can cause allergic or immunogenic reactions [20]. BNC has been used for dental and oral applications in bone tissue engineering primarily as a barrier material in guided tissue regeneration (GTR). The function of the cellulose membrane in this application is to guide the regeneration of the alveolar bone in periodontal diseases, allowing the bone cells to grow and form a functional tissue [21,22]. On the other hand, unmodified BNC has also been evaluated as a porous three-dimensional scaffold for bone regeneration [23] and as a scaffold to induce the differentiation of bone marrow mesenchymal stem cells (EqMSCs) into osteoblasts [24].

Bacterial nanocellulose has a low ability to bind directly to bone on its own; however, its versatility allows it to be modified with other materials, such as hydroxyapatite (HA), calcium phosphate (CaP) variants, or bioactive glasses, to increase its bioactivity and improve the process of bone regeneration [25]. Some scientific reports on two-dimensional, nonporous composites based on BNC/HA and BNC/CaP have shown that these composites have excellent moldability and biocompatibility properties for bone tissue regeneration applications [26,27]. In vitro, BNC has been shown to stimulate bone cell adhesion, migration, proliferation, and differentiation [28,29,30,31]. In one in vivo study, bone defects in rat tibiae were shown to be completely filled with new bone tissue 4 weeks after BNC introduction, with no inflammatory reaction [15]. The results of these investigations show that BNC has great potential as a bone graft material for the regeneration of hard tissues. However, more research is needed to design microporous 3D scaffolds that allow cell migration and proliferation. In addition, alternatives should be sought to increase the bioactivity of BNC, without affecting the morphology and interconnection of the pores in the scaffolds.

As mentioned above, BNC is structurally similar to the ECM of bone tissues because its nanofibrils are microscopically similar to collagen fibers [32]. In turn, the chemical characteristics of BNC (the presence of OH^−^ groups on the surface) allow for the creation of specific sites for the nucleation and growth of calcium phosphate crystals [33] through the interaction between Ca^2+^ ions and the OH^−^ groups of cellulose and the interaction with other ions that are naturally present in bone tissue, such as Na^+^, Mg^2+^, and K^+^ [34]. The objective of this research was to design a microporous scaffold based on bacterial nanocellulose, modified using a biomineralization method with alternating cycles of calcium and phosphate solutions, in order to mimic the structural and chemical characteristics of bone tissue. In addition, the scaffolds were chemically characterized to establish their characteristics and similarities with native bone. Finally, different in vitro tests were carried out to study cell viability and the expression of a series of specific markers that play different roles in the process of mesenchymal stem cell differentiation towards an osteoblastic phenotype [35,36,37].

## 2. Materials and Methods

### 2.1. Scaffold Preparation

**Figure 1:**
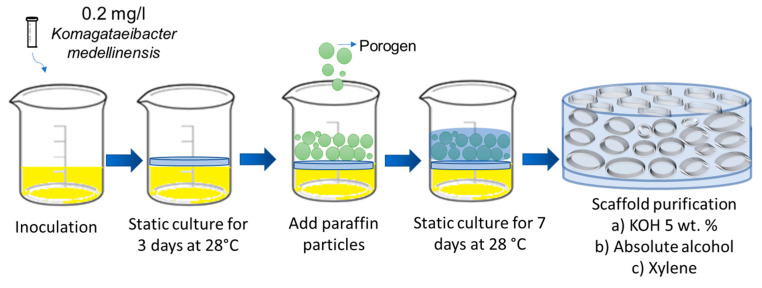
Schematic illustration of the design of bacterial nanocellulose microporous 3D scaffolds.

Bacterial nanocellulose (BNC) scaffolds were obtained through the fermentation of the bacterium *Komagataeibacter medellinensis* NBRC 3288 [38]. The strain was isolated at the Central Minorista de Medellín and was identified at the Universidad Pontificia Bolivariana [35]. The process of preparing the microporous 3D scaffolds is described in previous studies [36]. Briefly, nanocellulose scaffolds were prepared using a Hestrin–Schramm (HS) culture medium modified with 2% glucose (*w*/*v*), 0.5% peptone (*w*/*v*), 0.5% yeast (*w*/*v*), 0.267% disodium phosphate (*w*/*v*), and citric acid to adjust the pH to 3.5. The medium was inoculated with 0.2 mg/L of a *K. medellinensis* preinoculum. Fermentation was carried out initially for 3 days under static conditions to form a thin membrane that served as a scaffold for the paraffin microspheres. After this time, 0.2 g of paraffin microparticles was added to each well; the microparticles were prepared following the method reported by Osorio et al. [37], with a size between 50 and 350 µm. The fermentation was left for 7 more days until the nanocellulose covered the added paraffin microparticles. Then, the BNC scaffolds were purified in a 5% (*w*/*v*) KOH solution for 10 h at 100 RPM to remove biomass and debris. The scaffolds were then washed with distilled water until neutral pH was reached. Figure 1 shows a schematic illustration of the manufacturing process for the BNC microporous 3D scaffolds.

To eliminate the paraffin microspheres and obtain open pores, the BNC scaffolds were subjected to a series of washes with hydroalcoholic solutions of different concentrations (40, 60, and 100% (*v*/*v*)) in 2 h intervals at 75 °C. Afterwards, they were washed with xylene 2 times for 60 min each. Finally, another series of washes was carried out with hydroalcoholic solutions (100, 80, and 40% (*v*/*v*)). A cellulose membrane cultured for 7 days without the addition of paraffin particles was used as a control.

### 2.2. Modification of the Scaffolds

In the first treatment, the microporous 3D BNC scaffolds were immersed in a supersaturated solution of calcium chloride (CaCl_2_) at a pH of 7 for 48 h. Then, the scaffolds were treated with disodium phosphate solution (Na_2_HPO_4_) for 48 h. Throughout this time, the biomaterials were placed under dynamic conditions in an orbital shaker at 200 RPM and 37 °C. In the first treatment, the nucleation sites for the growth of calcium phosphate crystals were generated. Materials were prepared with 1, 2, 3, 4, and 5 cycles, designated as BNC CaP-X, where X corresponds to the number of cycles. The analyses used to determine the time required for calcium adsorption on the BNC surface are reported in previous studies [36].

### 2.3. Characterization of the Scaffolds

The morphology and microstructure of the microporous 3D BNC scaffolds, with and without the biomineralization process, were studied via scanning electron microscopy (SEM) using a JEOL JSM 6490 LV microscope (JEOL, Tokyo, Japan) in high vacuum with a secondary electron detector to obtain images of high-resolution SEI at an acceleration voltage of 20 KV and an EDS system. Cross sections of the scaffolds were freeze-dried and gold-sputtered in a Denton Vacuum Desk V (Moorestown, NJ, USA) for observation. The pore size of the scaffolds was measured using the freely distributed ImageJ software. In total, 100 surface pores were measured, taken from the SEM micrographs.

To assess the thermal decomposition of BNC and the relative proportions of the amount of mineral deposited on the surface of the material with the different treatment cycles, a thermogravimetric analysis (TGA) was performed using a Mettler Toledo TGA/SDTA 851E thermogravimetric analyzer (Mettler Toledo, Greifensee, Switzerland). The samples were left to dry in a forced convection oven at 105 °C until reaching a constant weight to obtain humidity control, considering the method described in Sampling and testing wood pulp shipments for moisture, Test Method T 210 cm-13. A total of 11 mg of each dry sample was used. The samples were heated from 30 to 800 °C at a heating rate of 10 °C min^−1^ in a nitrogen atmosphere.

The chemical composition of the biomineralized BNC microporous 3D scaffolds was analyzed by using energy dispersion X-ray spectroscopy (EDS) and X-ray fluorescence (XRF) on the surface of each dry scaffold. For XRF, a Thermo Scientific ARLTM Optim ‘x WDXDR spectrometer (Malvern, UK) was used in a helium atmosphere. For the EDS analyses, the chemical compositions of all scaffolds were analyzed using X-ray elemental maps in scan mode.

To detect the presence of the functional groups of the mineral and organic phases in the biomineralized scaffolds, Fourier transform infrared spectroscopy (FTIR) was carried out using a Thermo Scientific Nicolet 6700 spectrometer in attenuated total reflection (ATR) mode (Waltham, MA, USA). Spectra were recorded in the region between 4000 and 400 cm^−1^, using 64 scans and a spectral resolution of 6 cm^−^¹. Three measurements were made on each side of the scaffolds, and the spectra were averaged using the Omnic program.

X-ray diffraction (XRD) was used to determine the crystalline phases and the apparent crystal size of the mineral portion deposited on the scaffolds. The analysis was performed using a Rigaku X-ray diffractometer (Austin, TX, USA) with a copper (Cu) source. The diffractograms were obtained in the angular range of 5–50°, with a step size of 0.02°. The data were analyzed using the HighScore Plus Release software (Version 3.0d) to determine the crystalline phases of the biomineralized portion present in each modified scaffold through the search and match function [39,40]. The apparent crystal size (τ) was found using the Scherrer equation (Equation (1)) [4]:

(1) τ=Kλ/βcosθ

where ***K*** is the shape factor, for which a value of 0.94 was chosen (this value was used previously for elongated bone crystallites [4]); ***λ*** is the wavelength of the X-rays (0.15406 nm); ***θ*** is the Bragg angle; and ***β*** is the width at the mean height (FWHM) of the maximum intensity corresponding to the reflection of the (0 0 2) plane [40,41].

### 2.4. Cell Studies

In vitro biological assays were performed with human bone marrow mesenchymal stem cells (BM-MSCs), which were isolated from a bone marrow biopsy to obtain a primary cell culture. Signed informed consent was provided by healthy donors. The cells were cultured with Dulbecco′s modified Eagle′s culture medium (DMEM) media, with 10% FBS, 100 U/mL of penicillin, and 100 µg/mL of streptomycin, at 37 °C under 5% CO_2_. The BNC holders were sterilized in a LabTech autoclave for 20 min at 121 °C. Then, the materials were washed twice with a sterile phosphate-buffered saline solution (PBS) before a final wash was performed with DMEM.

The number of viable cells in direct contact with the scaffolds was determined by the formation of a colored compound through a reaction that takes place in the mitochondria of viable cells with the bromide of 3-(4,5-Dimethylthiazol-2-yl)-2,5-Diphenyltetrazolium Bromide (MTT). The cells were first seeded in 96-well plates at densities of 4.0 × 10^3^, 5.0 × 10^3^, and 6.0 × 10^3^ cells/well for specific time points of 24, 48, and 72 h. Then, 0.1 g/mL of the BNC scaffolds was incubated for the established times, following the international standard ISO 10993-5 2009. After each incubation period, the scaffolds were carefully removed, the culture medium was discarded, and 90 µL of fresh complete medium with 20 µL of 5 mg/mL MTT (Cayman Chemical, Ann Arbor, MI, USA) was added per well before incubation for 4 h at 37 °C. Hydrogen peroxide was used as a positive control with 100% toxicity, and wells with cells cultured without any material were used as a negative control. The test was carried out in triplicate.

The secretion activity of the alkaline phosphatase (ALP) enzyme was determined using an alkaline phosphatase detection kit, fluorescence (APF-1KT) (Sigma-Aldrich). The scaffolds were placed in 96-well culture dishes with 200 µL of DMEM medium, and cells were added at a density of 10 × 10^3^ cells/well. The scaffolds were incubated for 7 and 14 days at 37 °C in a humid atmosphere with 5% CO_2_. The culture media were conditioned to compensate for the loss of evaporated water from the dishes due to the long incubation time. After each timepoint, 180 µL of the solution prepared with the kit was added to each well. The absorbance was measured at a wavelength of 440 nm using a microplate reader (Bio-Rad). This assay was performed in triplicate. In addition, a fibroblast culture was used as a negative control and BM-MSCs as a positive control to determine the basal level of ALP.

The expression of osteopontin (OP) and osteocalcin (OC) was measured using the Western blot technique. To carry out this assay, an in vitro culture of BM-MSCs was first performed on the scaffolds using the same method explained above. After 21 days, the culture medium was removed from the wells and the cells were detached from the scaffolds with a trypsin solution. The obtained cells were lysed to release proteins using RIPA buffer (20-188, Sigma) supplemented with protease and phosphatase inhibitors. The proteins were loaded in 10 µg aliquots and separated in 14% (*w*/*v*) acrylamide gels mounted in an electrophoresis chamber at 180 V for 2 h at room temperature.

Then, the gel proteins were transferred to nitrocellulose membranes and placed in a chamber where a voltage of 40 V was applied for 2 h at 4 °C. The membranes were then incubated for 2 h in a blocking buffer and phosphate buffer solution with 4% (*w*/*v*) Tween 20 (PBST) before incubation with the primary antibodies (osteopontin AB10910 and osteocalcin AB 10911 Millipore) at a final concentration of 1 µg/mL in phosphate buffer–Tween 20 (PBST) for 12 h at 4 °C. Then, the membranes were incubated with a secondary antibody (IgG-HRP (AP160P, Millipore)) at a final concentration of 1 mg/mL in PBST. Finally, the membranes were viewed using a Luminata ™ Forte Western HRP transilluminator (WBLUF0100, Millipore). Unmodified BNC scaffolds cultured in medium from a StemPro ™ osteogenesis differentiation kit, which allows the complete differentiation of human mesenchymal stem cells into bone cells, were used as a positive control. Unmodified BNC scaffolds cultured in DMEM medium were used as a negative control. These tests were carried out at the Ramon Llull University, Barcelona, Spain.

Finally, statistical analyses were carried out where required using Statgraphics Centurion Version 2007. The data were analyzed with an analysis of variance (ANOVA) and the differences between means were determined using Fisher’s LSD multiple comparisons test. Data from test pairs that did not meet the assumptions for ANOVA were analyzed using a Kruskal–Wallis (K–W) test. A value of *p* ≤ 0.05 was considered statistically significant.

## 3. Results and Discussion

### 3.1. Scaffold Characterization

**Figure 2:**
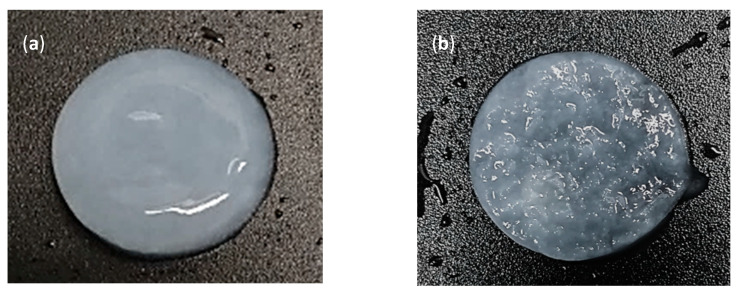

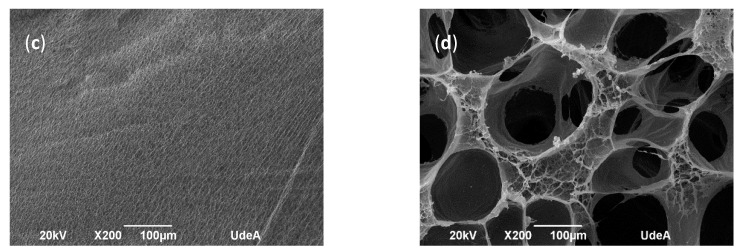
Scaffold morphologies. (**a**) SEM photograph and (**c**) micrograph of control BNC membrane. (**b**) SEM photograph and (**d**) micrograph of microporous 3D BNC scaffold.

The scaffolds were characterized through different physical and chemical analyses. Figure 2 shows photographs and electron microphotographs of the BNC membrane used as a control (Figure 2a,c) and the microporous 3D BNC scaffold (Figure 2b,d). From the photograph in Figure 2a, it can be seen that the surface of the nanocellulose membrane used as a control was more compact than the surface of the microporous material, which was more rough due to the paraffin particles (see Figure 2b). The electron micrographs show that the BNC scaffolds prepared with paraffin particles had a large number of pores, with a homogeneous distribution and a high level of interconnection between them (see Figure 2d). The pore sizes were between 70 and 350 µm. The pores maintained the spherical shape of the paraffin particles, suggesting that the process used to remove the paraffin from the BNC scaffolds was not aggressive and did not alter the 3D structure of the material. The results obtained up to this point are promising, since one of the key elements in bone tissue engineering is to manufacture a 3D scaffold with a porous and interconnected structure to allow cell migration and proliferation [23,42]. Some studies have shown that a pore size of 100 µm allows for cell migration, since the small pores favor hypoxic conditions and osteochondral formation. Other research suggests that pore sizes of around 300 µm easily allow revascularization, which favors osteogenesis. Therefore, the functional limit for pore size and distribution must first be established in order to regenerate bone properly [43,44]. Some studies have shown that porosity is a key characteristic of bone grafts since a high level of porosity and interconnectedness is important for vascularization and can promote osteoconductivity [41]. Therefore, it can be concluded that the pore size distribution obtained in the designed scaffolds will help the formation of new tissue.

**Figure 3:**
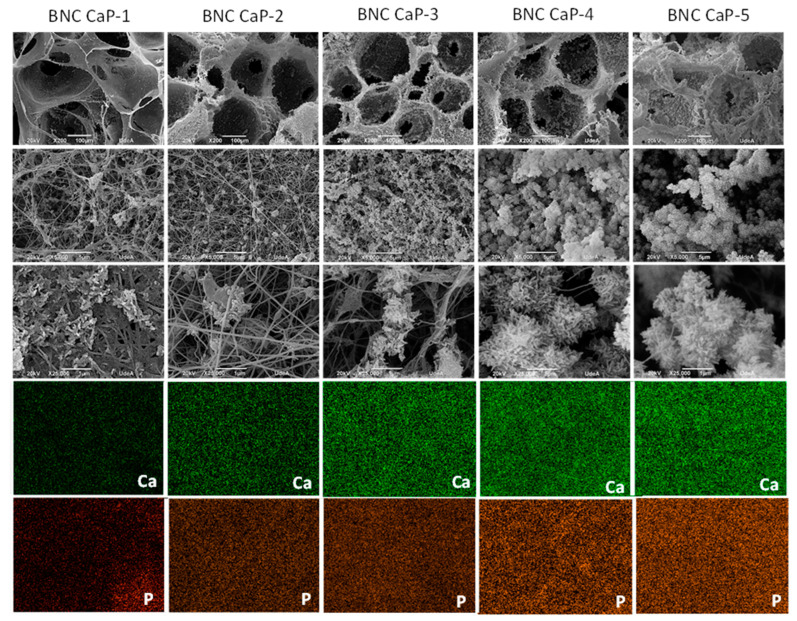
SEM micrographs taken at 200×, 5000×, and 25,000× (rows 1 to 3) and EDS elemental maps with a scale of 10 µm (rows 4 and 5) of the microporous 3D biomineralized BNC scaffolds (Ca corresponds to the element calcium and P corresponds to the element phosphate). From left to right: column 1—BNC cycle 1; column 2—BNC cycle 2; column 3—BNC cycle 3; column 4—BNC cycle 4; and column 5—BNC cycle 5.

Figure 3 shows the microporous 3D BNC scaffolds after the biomineralization cycles (BNC CaP-1 to BNC CaP-5). In the 200x micrographs, it can be seen that crystals were formed along the entire surface of the BNC pores and that the number and size of the crystals increased with an increasing number of cycles. The crystals around the cellulose nanofibers were shaped like bars or needles, as reported by other authors [15,45]. In the 5000× micrographs, it can be observed that, as the number of cycles increased, the distribution of the crystals was more homogeneous throughout the surface and larger crystallites were formed, as reported by other authors [45,46]. In the 200× micrographs, it can be seen that until the third cycle, the surface of the BNC was completely covered by the calcium phosphate crystals; however, the interconnectivity between the pores was still preserved (see Figure 3, BNC CaP-3 column). From the fourth and fifth treatment cycles, the crystals increased in size and quantity, closing the pores (see Figure 3, columns BNC CaP-4 and BNC CaP-5). Closed pores are not suitable for tissue engineering since, as mentioned earlier, scaffolds designed for tissue regeneration must have a network of interconnected pores to allow for cell migration, revascularization, and nutrient transport.

The homogeneous distribution of calcium and phosphate ions along the surface of the BNC from cycle one to cycle five can be observed in the EDS elemental maps in the lower part of Figure 3. The results obtained suggest that cyclical treatments with saturated CaCl_2_ solutions followed by Na2HPO4 solutions under dynamic conditions can simulate the mineralization conditions of bone tissues. Zimmermann et al. (2011), with this particular method, demonstrated that pretreatment with calcium allowed the creation of a nucleation site for the growth of calcium phosphate crystals [47].

**Figure 4:**
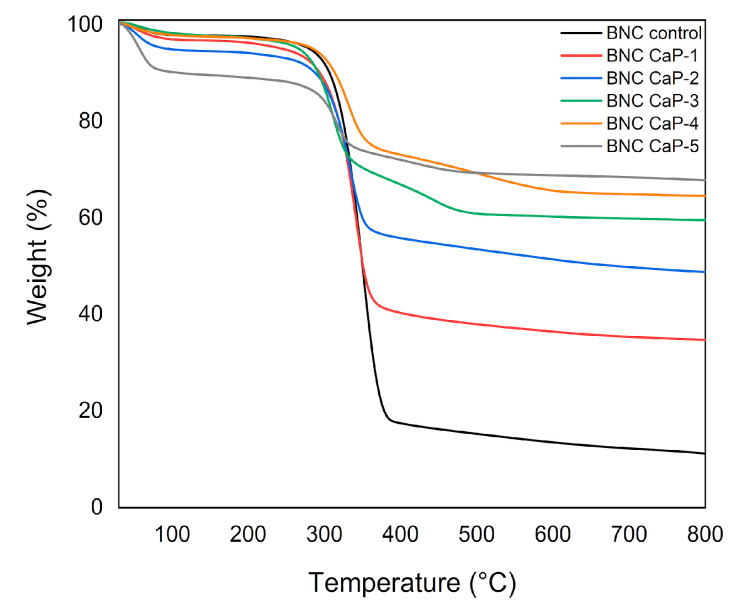
Results of the thermogravimetric analysis of biomineralized BNC microporous 3D scaffolds. An unmodified BNC microporous 3D scaffold was used as a control.

Figure 4 shows the thermograms obtained for the different materials. The results showed that, as the number of biomineralization cycles increased, the amount of mineral precipitated on the surface of the BNC scaffolds also increased. The percentage of solid waste by mass after 800 °C increased as follows: 34.8% for cycle one; 48.8% for cycle two; 59.6% for cycle three; 64.6% for cycle four; and 67.8% for cycle five. This increase was associated with the greater precipitation of calcium and phosphate ions with the increase in treatment cycles, as was also reported by Hutchens et al. (2006) [45]. This conclusion was corroborated by the electron micrographs shown in Figure 3, where the size and quantity of the crystals increased with the increase in the number of cycles. The mass loss rate of BNC was 11.3% due to charred ash in the nitrogen atmosphere [48].

Table 1 shows the results obtained for the mass fraction (% by weight) per chemical element using XRF. It can be observed that after the biomineralization cycles, Ca and P were the elements found in the highest proportions on the surface of the BNC, followed by Na. Calcium was the element with the highest concentration in all scaffolds; this is because BNC has a large amount of available –OH groups on carbon 6. When cellulose is introduced into media with a pH of 7, a negative surface charge is created on the –OH groups. This is what allows the adsorption of calcium ions by electrostatic interaction, as reported by other authors [33]. In addition, all hydroxyl groups in cellulose have negative dipoles that can chelate calcium ions (Ca^2+^) in solution and form a coordinated bond [36].

The results showed that the calcium phosphate ratio (Ca/P) increased with an increase in the number of cycles in the treatment. However, in all biomineralized scaffolds, this ratio was lower than that of stoichiometric hydroxyapatite (HA), which has a Ca/P ratio of 1.67. Hydroxyapatite is one of the most commonly used materials in bone grafts. However, it should be noted that the Ca/P ratio of bone increases with age and bone maturation, as confirmed by some reports in the literature [4,49]. This represents an advantage of the biomaterials designed in this work, since synthetic bone grafts can be manufactured with Ca/P ratios similar to those of natural bone, while taking into account the compositional variation in the tissue—which is associated with bone formation rates and bone resorption during the remodeling process—where Ca is found in greater quantities throughout the surface, since this ion plays a very important role in the formation of HA crystals in new bone [49]. Furthermore, Na^2+^ also plays an important role in bone crystal formation, as it often replaces Ca^2+^ in the crystal lattice of biological hydroxyapatite [34,49]. In addition to this, from Table 1 it can be seen that oxides such as CaO and P_2_O_5_ were also found, which are characteristic of mineralized bone.

**Figure 5:**
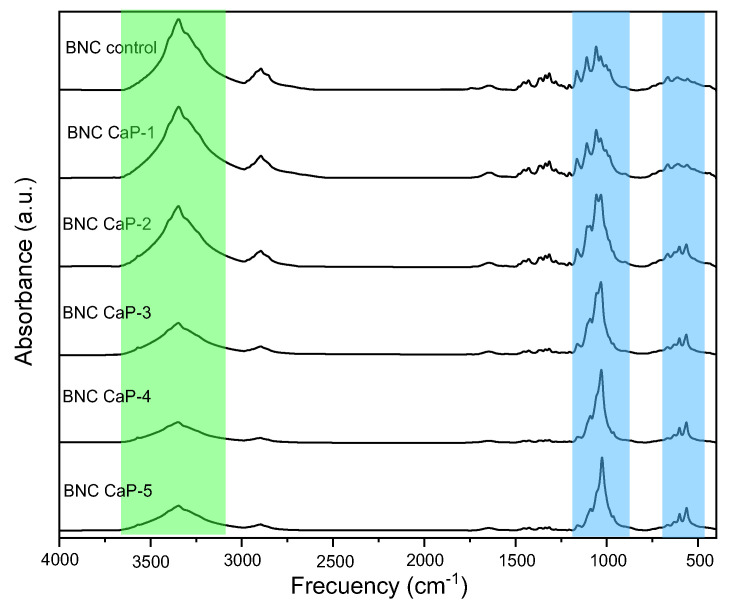
Analysis of the spectra obtained via FTIR-ATR of the biomineralized BNC microporous 3D scaffolds.

In Figure 5, the infrared spectra of the biomineralized BNC microporous 3D scaffolds can be observed with respect to the control BNC. The characteristic bands of BNC are found at ∼3350 cm^−1^ and are attributed to the stretching vibrations of the -OH group. The bands at ∼2890 cm^−1^, ∼1645 cm^−1^, ∼1440 cm^−1^**,** and ∼1065 cm^−1^ are attributed to the stretching vibrations of C-H and -CH_2_, the bending of –OH from adsorbed water, the symmetric bending of -CH_2_, and the skeletal vibrations of the pyranose ring C-O-C, respectively [50]. The bands at ∼1370 cm^−1^, ∼1340 cm^−1^**,** and ∼1315 cm^−1^ are attributed to the bending of -CH, the bending in the –OH plane, and the undulation of -CH_2_, respectively. This indicates the presence of crystalline regions within the structure of the BNC. Finally, the bands at ∼750 cm^−1^ and ∼710 cm^−1^ are related to the presence of crystalline Iα and Iβ allomorphisms of cellulose, respectively [44].

In Figure 5, it can also be observed that as the number of biomineralization cycles increased, the band at ∼3350 cm^−1^ attributed to the BNC began to disappear due to the cellulose being covered (Figure 5 green region)—as was also reported by other authors [33,51]—and the u_3_PO_4_^3−^ band appeared more defined and with greater intensity at ∼1200–900 cm^−1^ (indicated in the blue region of Figure 5), which is associated with calcium phosphates. This band became more defined and narrower as the number of cycles increased. The narrowness of this band is associated with the crystallinity of calcium phosphates [51,52]. In other words, the amount and crystallinity of the calcium phosphates precipitated on the BNC increased with the increase in the number of cycles. As the number of cycles increased, other characteristic bands also appeared, such as u_3_PO_4_^3−^, associated with phosphate ions that resolved into two peaks around 650–500 cm^−1^ [52,53] (marked in the blue region). The presence of this shoulder became more evident from cycle two to cycle five (BNC CaP 2-5). As reported in the literature, this may be indicative of the formation of octacalcium phosphate (OCP), which is the precursor phase in the formation of HA. As explained by Hutchens et al. (2006), the hydroxyl groups of cellulose have a strong negative dipole that can chelate the free Ca^2+^ cations in a CaCl_2_ solution and form a coordinate bond or an ion–dipole interaction. Phosphate ions can then bind with calcium already associated with cellulose to form calcium phosphate. Therefore, there is no initial direct interaction between calcium and phosphate; rather, the calcium ions are first ordered onto the BNC before mineralization, obtaining a phase that is not as crystalline as the OCP. However, increasing the number of cycles ensures a more crystalline and ordered apatite phase [45].

**Figure 6:**
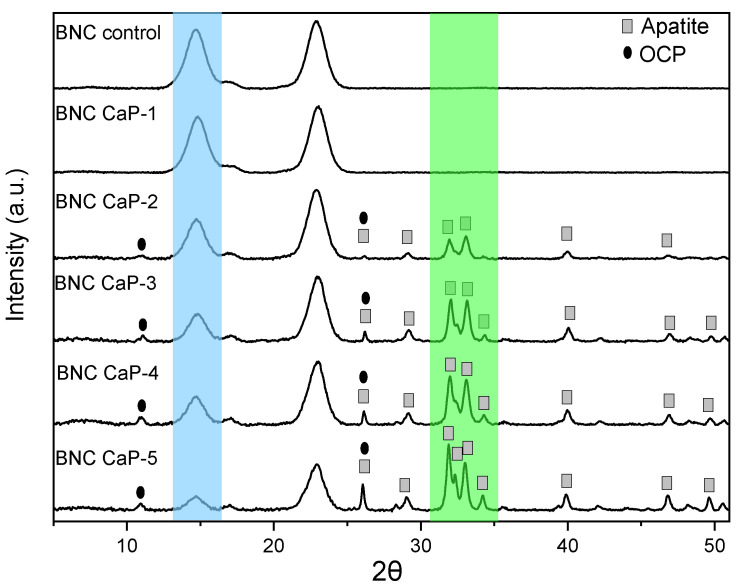
Phase analysis of the diffractograms of the biomineralized BNC microporous 3D scaffolds.

Figure 6 shows the diffractograms obtained from the biomineralized BNC microporous 3D scaffolds. All the scaffolds showed the three characteristic cellulose peaks assigned to the crystallographic planes (1 0 0), (0 1 0), and (1 1 0), which correspond to the diffraction angles of 14.6°, 16°, and 22.8°, respectively. In the blue region indicated in Figure 6, it can be observed that, as the amount of minerals deposited on the surface of the scaffolds increased, the intensity of the BNC peaks decreased. The decrease in the intensity of these peaks shows that as the microporous 3D scaffolds were continually modified by the biomineralization treatment, the mineral phases became the dominant component of the scaffold due to the increase in the amount of calcium phosphates. This was also confirmed by studies carried out by Hutchens et al. (2006) and Yin et al. (2011) investigating mineralization processes on cellulose membranes [45,50].

Therefore, for the semiquantitative phase analysis using the HighScore software, the mineral elements found via XRF were used as established restrictions, and the characteristic peaks of cellulose were eliminated. From the phase analysis of the microporous 3D BNC scaffolds, it was found that with one treatment cycle, no characteristic mineral phase was observed. Only the treatments with two to four cycles resulted in phases of octacalcium di-hydrogen phosphate pentahydrate (OCP) and apatite-(CaOH). With five cycles, the apatite phase was predominant (see green region of Figure 6). Table 2 shows the semiquantitative results obtained from the HighScore software.

This information is correlated with the diffractograms in Figure 6, where it can be observed that there was an increase in the crystalline peaks associated with the OCP and apatite phases found in the biomineralized scaffolds from cycle two and onwards. The main diffraction peaks of the apatite phase of the scaffolds were 25.86°, 28.73°, 31.7°, 32.14°, 32.8°, 34.01°, 39.67°, 46.57°, and 49.41°, which correspond to the diffraction planes (0 0 2), (1 0 2), (2 1 1), (1 1 2), (3 0 0), (2 0 2), (3 1 0), (2 2 2), and (2 1 3), respectively [40,54]. The diffraction peaks for the OCP phase of the scaffolds were found at 26.37° and 32.56°, corresponding to the diffraction planes (0 0 2) and (7 0 0), respectively [55]; in addition, they were more evident in the BNC scaffolds from the third treatment cycle onwards. These mineral phases correspond to the Ca/P ratios found in the materials using XRF, which were between 1.19 and 1.60. These two phases of calcium phosphates are predominant in human bones, which indicates that this biomineralization method allows for the design of materials with great similarity to the mineral phases of bone.

Finally, from the second cyclic treatment onwards, the biomineralized BNC microporous 3D scaffolds showed the presence of a crystallographic plane at (0 0 2), corresponding to a diffraction angle at 26.3°, which is associated with both the apatite phase and the OCP phase. From the width at the mean height (FWHM) of the maximum intensity corresponding to the reflection of plane (0 0 2), the apparent size of the crystals on the different biomineralized scaffolds was determined [27,40,41]. Table 3 shows the values for apparent crystal size from the second to the fifth cycle. The results show that, as the number of cycles in the biomimetic treatment increased, the apparent size of the crystals also increased. The apparent size of the crystals found in these scaffolds is within the range of the sizes reported in the literature [45,51]. These results are consistent with what was observed in the micrographs in Figure 3, where larger crystals were formed as the number of cycles increased.

### 3.2. Cell Studies

**Figure 7:**
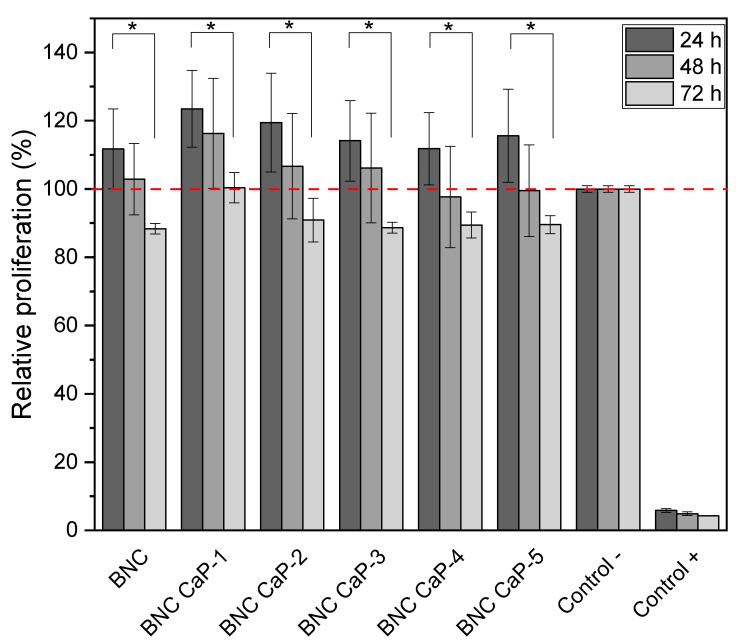
Viability of BM-MSCs cultured on biomineralized BNC microporous scaffolds. Hydrogen peroxide was used as positive control and cells grown in a dish were used as negative control. * Significant difference (*p* < 0.05) at a confidence level of 95% (*n* = 3). Data were analyzed using a one-way ANOVA test.

The in vitro behavior of the scaffolds was determined via cytotoxicity tests and APL, OP, and OC protein expression in BM-MSCs, which are well described as precursor cells of osteoblasts [11]. Figure 7 shows the results obtained from the MTT assay after 24, 48, and 72 h of BM-MSC cell culture. During the first 24 and 48 h, cell viability remained at 100%, and no statistically significant differences (*p* ˃ 0.05) were observed between the different BNC scaffolds and the unmodified BNC used as a control (see the red dotted line in Figure 7). After 72 h of culture, all the designed scaffolds presented a statistically significant difference (*p* ˂ 0.05) in the percentage of cell viability with respect to the culture after 24 h. However, cell viability remained above 90% for all scaffolds. The decrease in the percentage of cell viability at 72 h could be explained by the increase in Ca^2+^ concentration in the culture medium, caused by the release of calcium ions from the scaffolds that were initially treated with supersaturated calcium solutions. As reported by Lee et al. (2018), adequate Ca^2+^ concentrations in the culture medium improve cell adhesion, proliferation, and differentiation; however, if they are very high in the assay microenvironment (culture dishes), they can be toxic to cells [56]. Therefore, future research should focus on the microenvironment generated by biomineralized BNC scaffolds and whether or not there is release of ions associated with calcium phosphate crystals into the culture medium over time.

Another important factor to consider is the depletion of nutrients in the growth medium over time. The designed scaffolds, being microporous, have a much greater surface area than the flat surface of the culture dish that was used as a negative control. Therefore, there was a considerable initial increase in cell density, as can be seen in Figure 7, where viability was higher than in the negative control (red dotted line) after 24 h. In addition, in most cases, it was maintained after 48 h. In general, these cell viability results suggest that the protocols designed for the preparation of the microporous 3D scaffolds, the washes used to remove the porogenic agents from paraffin, the biomineralization methods, and the nature of the materials do not generate any cytotoxic damage to cells and, on the contrary, promote a viable and proliferative cell culture.

**Figure 8:**
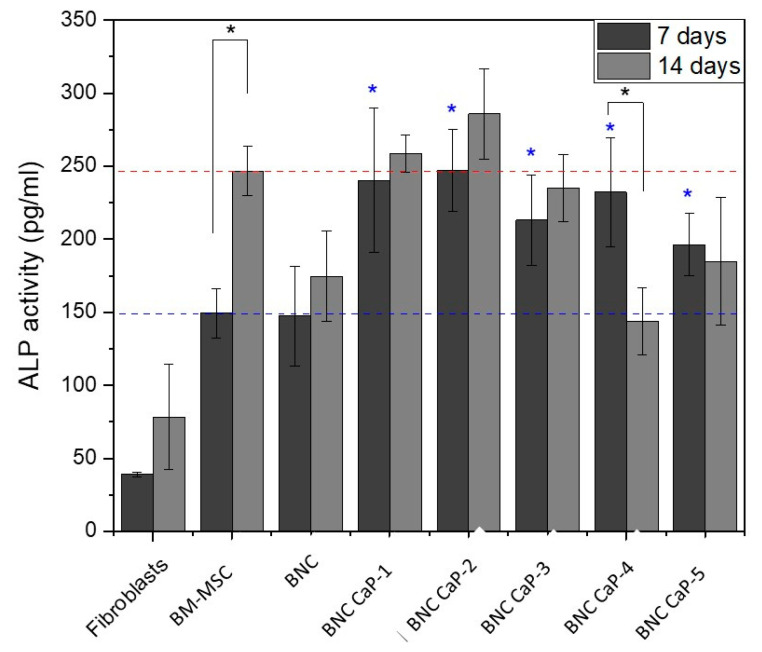
Expression of ALP in BM-MSCs cultured on biomineralized BNC microporous scaffolds for 7 and 14 days. A culture of fibroblasts, which are cells that do not express ALP, was used as negative control, while BM-MSCs were used as positive control to determine the basal level of ALP. * Significant difference (*p* < 0.05) at a confidence level of 95% (*n* = 3). Data were analyzed using a Kruskal–Wallis test.

Figure 8 shows the expression of ALP after 7 and 14 days for each of the designed BNC scaffolds, including the unmodified BNC and the controls. Alkaline phosphatase (APL) plays an important role in the bone mineralization process and is used as an early marker for the differentiation of BM-MSCs into osteoblasts (Sharma et al., 2014). After 7 days of culture, a statistically significant difference (*p* < 0.05) in ALP expression was observed between the BM-MSCs incubated with BNC scaffolds and the control cells (see blue asterisks). All cells incubated with the BNC scaffolds exhibited higher levels of ALP expression (blue dotted line). BM-MSCs alone were used to analyze the basal level of ALP without stimulation. No statistically significant differences in ALP expression were found (*p* ˃ 0.05) between BM-MSCs cultured on the plate and BM-MSCs cultured on BNC scaffolds without any biomineralization treatment. As reported by Shi et al. (2012), BNC alone does not favor the expression of ALP in amounts comparable to the baseline level of MSCs [57].

On the other hand, after 14 days of culture, a higher expression of ALP activity was obtained in the BNC CaP-1, BNC CaP-2, and BNC CaP-3 scaffolds when compared to the basal level in BM-MSCs. In the other BNC scaffolds, a decrease in ALP was observed between 7 and 14 days of culture. A possible explanation for this decrease in ALP is based on the fact that the increase in this enzyme during the first 7 days is an indicator of the active differentiation of BM-MSCs towards preosteoblasts [11,58], allowing the creation of nucleation sites for the mineralization of bone tissue. Subsequently, as reported by various authors, after the start of mineralization, ALP is no longer needed; therefore, the cellular levels of the enzyme decrease before a mature mineralized matrix is formed. Thus, the decrease in the expression of ALP may be indicative of the initiation of the biomineralization of preosteoblasts on the BNC scaffolds [59,60]. The results for ALP expression in BM-MSCs grown on the different biomineralized BNC scaffolds indicate that biomineralized BNC scaffolds do promote the secretion of this protein by BM-MSCs.

**Figure 9:**
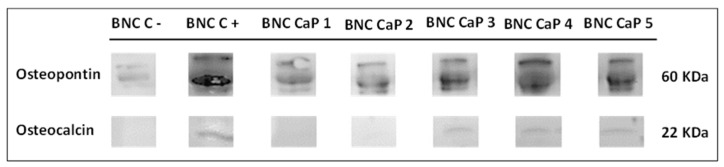
Expression of OP and OC in BM-MSCs cultured on biomineralized BNC microporous scaffolds for 21 days. As a positive control, a culture with unmodified BNC scaffolds in a medium that allowed the complete differentiation of human mesenchymal stem cells into bone cells was used. Unmodified BNC scaffolds in DMEM culture medium were used as a negative control.

Finally, the expressions of osteopontin (OP) and osteocalcin (OC) were determined in order to follow the process of BM-MSC differentiation towards osteoblasts. Figure 9 shows the results obtained via Western blotting for the expression of OP and OC in the BM-MSCs grown on biomineralized BNC microporous scaffolds after 21 days of culture. In all the scaffolds, the expression of OP was observed with three bands, where the thickest and most intense band was seen around 60 kDa (the molecular weight associated with the characteristics of the primary antibody used). An increase in the intensity of OP expression was also observed as the number of cycles increased. The BNC scaffold without any biomineralization showed a very low intensity of OP expression compared to the biomineralized BNC scaffolds. As mentioned above, the BNC CaP-4 and BNC CaP-5 scaffolds showed a decrease in ALP after 14 days of culture, as shown in Figure 8. Taking these results together, it can be suggested that these treatments may have accelerated the maturation process of the preosteoblasts towards osteoblasts. In general, the expression of OP in all biomineralized BNC scaffolds indicates the maturation process of preosteoblasts towards osteoblasts [56] when compared with the positive control (which consisted of BM-MSCs cultured on unmodified BNC scaffolds in a culture medium that allowed for the complete differentiation of human mesenchymal stem cells into bone cells) and negative control. This result also suggests that the designed scaffolds have a high osteoinductive potential, since during the process of new bone formation, this protein adheres to the surface of the mineralized material, helping to activate the cellular signaling pathways that attract osteoblast progenitors and regulate mineralization by improving the material–bone interface [61]. In addition, due to the RGD (arginine–glycine–aspartic acid) domains in its amino acid sequence, the presence of this protein improves cell adhesion, promotes bone formation through the intra- and extracellular regulation of Ca^2+^, and stimulates bone resorption by possessing chemotactic activity for osteoclasts [56,62].

On the other hand, Figure 9 shows that after 21 days of culture, only the 22 kDa OC protein was expressed on the BNC CaP 3, BNC CaP 4, and BNC CaP 5 scaffolds. OC is a specific protein in bone synthesized only by mature osteoblasts and involved in the binding of calcium and hydroxyapatite [11,63]. Therefore, the presence of this protein in the BM-MSC cultures on the aforementioned scaffolds suggests that these materials favored osteoblastic differentiation [64,65] (Ferreira et al., 2012; Sun et al., 2012) after 21 days of culture when compared to the other BNC scaffolds.

## 4. Conclusions

The results obtained showed that, as the number of cycles in supersaturated solutions increased, mineral deposition increased, and the amount and size of calcium phosphate crystals on the surface of the microporous 3D BNC scaffolds also increased. This affected the size and interconnection between the pores, which could hamper the tissue formation process. For this reason, it is advisable to carry out a biomineralization treatment of up to three cycles.

The in vitro cultures demonstrated that the materials and processes designed to manufacture the three-dimensional scaffolds did not affect cell viability. In addition, through the detection of specific cell markers for the process of bone maturation in vivo, it was shown that the biomineralized BNC microporous 3D scaffolds generally favored the differentiation of BM-MSCs towards osteoblasts. This was demonstrated by the early expression of alkaline phosphatase (ALP), which indicates the differentiation of BM-MSCs into preosteoblasts. Then, a high expression of osteopontin (OP) was observed, which indicates the continuation of the preosteoblast maturation process and marks the beginning of mineralization. The expression of osteocalcin (OC) was detected only in the BNC CaP-3 scaffolds, which is an indicator that these materials could possibly accelerate the maturation process in vivo and increase the degree of bone mineralization compared to the other scaffolds, which were also shown to favor the differentiation process. These results are promising since the differentiation behavior of the designed BNC scaffolds was found to be analogous to what occurs in the maturation and remodeling process of bone.

## Figures and Tables

**Table 1:** Results of the elemental analysis using X-ray fluorescence.

Scaffold *	Ca	P	Na	Mg	S	K	Cl	Fe	Si	CaO	P_2_O_5_	Ca/P (Atomic %)
NCB CaP-1	10.33	7.09	1.01	-	0.03	0.01	0.10	0.02	-	19.34	20.01	1.13
NCB CaP-2	16.90	10.94	1.11	-	0.45	0.07	0.20	0.04	-	23.64	23.69	1.19
NCB CaP-3	20.74	12.95	0.68	-	0.03	0.01	0.07	0.03	-	29.01	29.67	1.24
NCB CaP-4	22.16	14.01	1.22	-	0.03	0.01	0.05	0.03	0.01	31.00	32.10	1.22
NCB CaP-5	26.61	12.82	0.82	-	0.02	0.02	0.16	0.04	0.03	37.22	29.83	1.60

* One gram of sample (% mass) of the biomineralized BNC microporous 3D scaffolds and Ca/P ratio atomic %.

**Table 2:** Results of semiquantitative mineral phase analyses performed with the HighScore Plus Release software (Version 3.0d).

Scaffold	Phase	Semiquantitative %
NCB CaP-1	Calcium	98
NCB CaP-2	Calcium	14
	Apatite	28
	OCP	58
NCB CaP-3	Calcium	8
	Apatite	22
	OCP	71
NCB CaP-4	Calcium	8
	Apatite	24
	OCP	78
NCB CaP-5	Calcium	5
	Apatite	95

**Table 3:** Results for the apparent crystal size in plane (0 0 2) of the 3D microporous BNC scaffolds.

	Apparent Crystal Size in Crystallographic Plane (0 0 2)		
Scaffold	2θ	(τ) nm	d (nm)
BNC CaP 2	26.15	25.12	0.34
BNC CaP 3	26.21	34.70	0.34
BNC CaP 4	26.14	36.12	0.34
BNC CaP 5	26.06	35.90	0.34
HA *	25.81	54.36	0.34

* Commercial hydroxyapatite taken as reference.

## Data Availability

The data presented in this study are available on request from the corresponding author.
